# Identification and functional analysis of two Golgi-localized UDP-galactofuranose transporters with overlapping functions in *Aspergillus niger*

**DOI:** 10.1186/s12866-015-0541-2

**Published:** 2015-11-02

**Authors:** Joohae Park, Boris Tefsen, Marc J. Heemskerk, Ellen L. Lagendijk, Cees A. M. J. J. van den Hondel, Irma van Die, Arthur F. J. Ram

**Affiliations:** Leiden University, Institute of Biology Leiden, Molecular Microbiology and Biotechnology, Sylviusweg 72, 2333 BE Leiden, The Netherlands; Department of Molecular Cell Biology and Immunology, VU University Medical Center, van den Boechorststraat 7, 1081 BT Amsterdam, The Netherlands; Department of Biological Sciences, Xi’an Jiaotong-Liverpool University, 111 Ren Ai Road, Dushu Lake Higher Education Town, Suzhou Industrial Park, Suzhou, Jiangsu 215123 China

**Keywords:** Cell wall, Galactomannan, Galactofuranose, Sugar nucleotide transporters, Golgi

## Abstract

**Background:**

Galactofuranose (Gal*f*)-containing glycoconjugates are present in numerous microbes, including filamentous fungi where they are important for morphology, virulence and maintaining cell wall integrity. The incorporation of Gal*f*-residues into galactomannan, galactomannoproteins and glycolipids is carried out by Golgi-localized Gal*f* transferases. The nucleotide sugar donor used by these transferases (UDP-Gal*f*) is produced in the cytoplasm and has to be transported to the lumen of the Golgi by a dedicated nucleotide sugar transporter.

**Methods:**

Based on homology with recently identified UDP-Gal*f*-transporters in *A. fumigatus* and *A. nidulans*, two putative UDP-Gal*f*-transporters in *A. niger* were found. Their function and localization was determined by gene deletions and GFP-tagging studies, respectively.

**Results:**

The two putative UDP-Gal*f*-transporters in *A. niger* are homologous to each other and are predicted to contain eleven transmembrane domains (UgtA) or ten transmembrane domains (UgtB) due to a reduced length of the C-terminal part of the UgtB protein. The presence of two putative UDP-Gal*f*-transporters in the genome was not unique for *A. niger.* From the twenty Aspergillus species analysed, nine species contained two additional putative UDP-Gal*f*-transporters. Three of the nine species were outside the *Aspergillus section nigri*, indication an early duplication of UDP-Gal*f*-transporters and subsequent loss of the UgtB copy in several aspergilli. Deletion analysis of the single and double mutants in *A. niger* indicated that the two putative UDP-Gal*f*-transporters (named UgtA and UgtB) have a redundant function in UDP-Gal*f*-transport as only the double mutant displayed a Gal*f-*negative phenotype. The Gal*f*-negative phenotype of the double mutant could be complemented by expressing either CFP-UgtA or CFP-UgtB fusion proteins from their endogenous promoters, indicating that both CFP-tagged proteins are functional. Both Ugt proteins co-localize with each other as well as with the GDP-mannose nucleotide transporter, as was demonstrated by fluorescence microscopy, thereby confirming their predicted localization in the Golgi.

**Conclusion:**

*A. niger* contains two genes encoding UDP-Gal*f*-transporters. Deletion and localization studies indicate that UgtA and UgtB have redundant functions in the biosynthesis of Gal*f*-containing glycoconjugates.

**Electronic supplementary material:**

The online version of this article (doi:10.1186/s12866-015-0541-2) contains supplementary material, which is available to authorized users.

## Background

The cell wall is an important extracellular structure of fungal species. It is essential for growth and survival as it protects the cell from lysis by its internal turgor pressure. The cell wall represents a significant investment of the cell as about 30 % of the cellular dry weight consists of this dynamic barrier [1]. The cell wall of filamentous fungi is composed of several different carbohydrate polymers (chitin, β-1,3-glucan, β-1,3/1,4-glucan, α-glucan, galactosaminogalactan and galactomannan) and glycoproteins (galactomannoproteins) [2]. The galactose in galactomannan and galactomannoproteins in filamentous fungi is present in the form of galactofuranose (Gal*f*), the five-membered ring form of this hexose. In contrast, the galactose found in galactomannoproteins in yeasts such as *Saccharomyces cerevisiae* and *Schizosaccharomyces pombe* and galactosaminogalactan in aspergilli is the more common galactopyranose (six-membered ring form; Gal*p*) [3]. Because the presence of Gal*f* is often associated with virulence in pathogenic bacteria, fungi and protozoan, it is considered as an important virulence factor and its biosynthesis as a target for antibiotics [4–7].

Many of the cell wall polymers (e.g. chitin, β-1,3-glucan, and α-glucan) are synthesized by plasma membrane localized enzymes that use nucleotide sugars UDP-*N*-acetylglucosamine (chitin), UDP-glucose (β-1,3-glucan, α-glucan)) as sugar donors (see [8] for recent review). In contrast, galactomannan, including its Gal*f* side-chain, is stepwise assembled during their transit through the secretory pathway [9, 10]. The localization of the biosynthesis of galactomannan is not known in detail but is likely to involve the synthesis of a galactomannan-GPI-anchor precursor in the ER, whereafter it is further elongated in the Golgi by specific mannosyltransferases and Gal*f* transferases [10]. The mannosyltransferases involved in the incorporation of mannose residues on galactomannan in the ER or the Golgi are currently not known. The Gal*f* transferase genes (*Gfs*) have recently been identified in *A. fumigatus* and *A. nidulans* [10]. Transfer of activated sugar-nucleotides from the cytoplasm to the lumen of the Golgi is an essential requirement for glycosylation of glycoproteins and carried out by sugar-nucleotide specific transporters for GDP-mannose [11–13] and UDP-galactose [14], respectively. Golgi-localized nucleotide sugar transporters are structurally conserved Type III transmembrane proteins, but little is known regarding their exact structure due to the difficulty to crystallize membrane proteins. In general, nucleotide sugar transporters contain an even number of transmembrane domains (6, 8, or 10) with both the N-terminus and the C-terminus present on the cytosolic side (see for a recent review by Hadley [15]). The GDP-mannose transporter is relatively well characterized in *S. cerevisiae* [13, 16] as well as in Aspergilli [11, 12, 17]. GFP-tagging of GDP-mannose transporters in *A. niger* and *A. nidulans* have shown the typical Golgi localization of the GDP-mannose transporter [11, 12]. An UDP-Gal*f* transporter was first identified and characterized in *A. fumigatus* [18] and subsequently also in *A. nidulans* [19]. Fractionation studies indicated Golgi localization of the transporter, which was further confirmed in *A. nidulans* by FLAG-tagging [18]. In this paper, we show that *A. niger* possesses not a single, but two genes encoding UDP-Gal*f* transporters. Both genes are functional and the two proteins have redundant functions as deletion of both genes is required to block galactofuranosylation.

## Methods

### Strains and growth conditions

The *A. niger* strains used in this study are listed in Table 1. Strains were grown on minimal medium (MM) [20] containing 1 % (wv-1) glucose as carbon source or on complete medium (CM) containing 0.5 % (wv-1) yeast extract and 0.1 % (wv-1) casamino acids in addition to MM. When required, plates were supplemented with 10 mM uridine. 5’FOA selection for the selection of *pyrG*^−^ strains was performed as described previously [24]. For the plate growth assays, strains were grown on CM or MM plates supplemented with 0.0025 % SDS or CFW as described [25].

**Table 1:** Strain used in this study

Strain	Genotype	Relevant genotype	Reference
N402	*cspA*	wild type	[21]
MA70.15	*ΔkusA::amdS*, *pyrG* ^*−*^	wild type	[22]
MA169.4	*ΔkusA::DR-amdS-DR pyrG* ^*−*^	wild type	[23]
MA234.1	*ΔkusA::DR-amdS-DR pyrG* ^*+*^	wild type	[26]
MA87.6	*ΔugmA* in MA70.15	*ΔugmA*	This study
JP9.1	*ΔugtA::hygB* in MA234.1	*ΔugtA*	This study
JP10.1	*ΔugtB::pyrG* in MA169.4	*ΔugtB*	This study
JP11.1	*ΔugtA::hygB* in JP10.1 (Δ*ugtB*)	*ΔugtAΔugtB*	This study
MH1.1	*ugtA-CFP in MA169.4*	*ugtA-CFP*	This study
MH2.1	*ugtB-CFP in MA169.4*	*ugtB-CFP*	This study
MH3.1	*pyrG-* derivative of MH1.1	*ugtA-CFP, pyrG-*	This study
MH4.1	*pyrG-* derivative of MH2.1	*ugtB-CFP, pyrG-*	This study
MH5.1	*ΔugtA::hygroB* in MA169.4	*ΔugtA*, *pyrG-*	This study
MH6.1	*ugtB-CFP(pyrG+)* in MH5.1	*ugtB-CFP i*n *ΔugtA*	This study
MH7.1	*ugtA-CFP (pyrG+)* in MH9.1	*ugtA-CFP* in *ΔugtB*	This study
JH24.3	*ugtB-YFP (pyrG+)* in MH3.1	*ugtA-CFP*, *ugtB-YFP*	This study
MH9.1	*pyrG-* derivative of JP10.1	*ΔugtB, pyrG-*	This study
JH22.3	GmtA-YFP in MH3.1	*ugtA-CFP*, *gmtA-YFP*	This study
JH23.3	GmtA-YFP in MH4.1	*ugtB-CFP*, *gmtA-YFP*	This study

### General molecular techniques

*Escherichia coli* DH5α strains were transformed by electroporation for propagation and amplification of the plasmids. Amplification of plasmid DNA was performed using the XL1-Blue strain, which was transformed using the heat-shock protocol as described by Inoue [26]. Transformation of *A. niger* and isolation of genomic DNA was performed as described by Meyer [27]. [α-^32^P]dCTP-labeled probes for Southern blots were synthesized using the Rediprime II DNA labeling system (GE Healthcare Life Sciences) according to the instructions of the manufacturer. All molecular techniques including cloning and PCR amplifications and Southern blotting were carried out as described by Sambrook [28]. DNA sequencing was performed by Macrogen Europe (Amsterdam, The Netherlands).

### Construction of *ugtA* and *ugtB* deletion and fluorescent protein tagged strains

For the construction of the *ugtA* and *ugtB* deletion strains deletion cassettes were made using the Multisite Gateway® Three-Fragment Vector Construction kit. As a marker for deleting the *ugtB* gene, the *pyrG* marker of *A. oryzae* was used. To facilitate removal of the *AopyrG* marker, *A. nidulans* tTrpC repeats were included around the *pyrG* gene [24]. The hygromycin B (*HygroB*) selection marker was used for the deletion of the *ugtA* gene. All flanking regions of *ugtA* and *ugtB* as well as the selection markers were PCR amplified using the primers listed in Supplemental Table 1 and cloned in appropriate pDONR vectors. For all the amplifications Phusion™ High-Fidelity DNA polymerase was used (Finnyzymes®) and gDNA from *A. niger* strain N402 was used as template DNA to amplify the fragments. The subsequent LR reaction was performed using the three pDONR vectors and pDEST R4-R3 Vector 2 to create the *ugtA and ugtB* deletion plasmids. The final constructs were verified by restriction analysis and sequencing.

The construction plasmids expressing FP-labeled transporters (UgtA-CFP, UgtB-CFP and UgtB-YFP) were also made using the Gateway® Three-Fragment Vector construction kit. Fragments containing the *ugtA* 5’ ORF (942 bp), the *ugtA* 3’flank (1040 bp), *ugtB* 5’ ORF (876 bp) and *ugtB* 3’ flank (1008 bp) were amplified using primers listed in Table 2. The amplified fragments used in a BP-reaction to give various Donor vectors. The resulting pDonR vectors were: pDonR-UgtA5’, pDonR-UgtA3’, pDonR-UgtB5’ and pDonR-UgtB3’. pDonR-CFP-TPT and pDonR-YFP-TPT (courtesy of Benjamin Nitsche) bearing the fluorescent protein and the *tTrpC*-*pyrG*-*tTrpC* (TPT) selection marker fused to either CFP or YFP were used in the LR reaction. Appropriate pDonR fragments were recombined in the LR reaction to give pDEST vectors MJ1.1 (UgtA-CFP), MJ2.1 (UgtB-CFP) and MJ3.1 (UgtB-YFP). The pDEST vectors were sequenced and subsequently transformed in *A. niger* strain MA169.4 (*ΔkusA*, *pyrG*^*−*^). Transformants were purified on MM and checked in a Southern Blot. Transformants containing a single copy of UgtA-CFP, UgtB-CFP or UgtB-YFP at the *ugtA* or *ugtB* locus were selected for further analysis. For the construction of double mutants, the *pyrG* marker of removed by isolating 5’FOA resistant strains as described [24]. 5’FOA resistant mutants in which the *pyrG* gene had been looped out were used to construct UgtA-CFP/UgtB-YFP, UgtA-CFP/GmtA-YFP, and UgtB-CFP/GmtA-YFP double fluorescent strains (Table 1). Targeted integration of GDP-mannose transporter (GmtA) at the *pyrG* locus using the pyrG* allele was done using the construct described previously [12].

**Table 2:** Primers used in this study

Primer	Sequence (5’-3’)^a^	Description
attB4_UDPgalFT5F	ggggacaactttgtatagaaaagttgAGAAACTTTAGCCAGAACTT	*ugtA* 5’ element
attB1r_UDPgalFT5R	ggggactgcttttttgtacaaacttgAGGACTGACTAGAAGTTCAG	*ugtA* 5’ element
attB2r_UDPgalFT3F	ggggacagctttcttgtacaaagtggTGTGAAAGTGCGAGTCTGAT	*ugtA* 3’ element
attB3_UDPgalFT3R	ggggacaactttgtataataaagttgGGATTCAGGTCCGGGTCCAG	*ugtA* 3’ element
attB4_ugtB5F	ggggacaactttgtatagaaaagttgGGTCGACCAGACTCCACCAA	*ugtB* 5’ element
attB1r_ugtB5R	ggggactgcttttttgtacaaacttgGATGGACGGTCGCACACGAG	*ugtB* 5’ element
attB2r_ugtB3F	ggggacagctttcttgtacaaagtggAGTCGATTGTACATATGGTA	*ugtB* 3’ element
attB3_ugtB3R	ggggacaactttgtataataaagttgGCGACAAGAACACCATTGGC	*ugtB* 3’ element
attB4 FW_ugtA5	ggggacaactttgtatagaaaagttgTGGTCAGTCATTCCCTTTCGAGC	*ugtA*_CFP_tagging
attB1 R_ugtA5	ggggactgcttttttgtacaaacttgAGGCATTTCCAGCAGTAGCGC	*ugtA*_CFP_tagging
attB2 FW_ugtA3	ggggacagctttcttgtacaaagtggAGGACTGACTAGAAGTTCAGG	*ugtA*_CFP_tagging
attB3 R_ugtA3	ggggacaactttgtataataaagttgACCGTACAGTAACAGGTGAC	*ugtA*_CFP_tagging
attB4 FW_ugtB5	ggggacaactttgtatagaaaagttgTGACCTCAGTGTGTCCTTCATCC	*ugtB*_CFP_tagging
attB1 R_ugtB5	ggggactgcttttttgtacaaacttgAGTTCTCGGGGCGGGGGCCAC	*ugtB*_CFP_tagging
attB2 FW_ugtB3	ggggacagctttcttgtacaaagtggAGTCGATTGTACATATGGTA	*ugtB*_CFP_tagging
attB3 R _ugtB3	ggggacaactttgtataataaagttgTACCACCTTCACCCTTGACC	*ugtB*_CFP_tagging

^a^ nucleotides in lowercase indicate attB recombination sites

### Fluorescent microscopy

Light and fluorescence microscopic pictures were captured with a 63x C-apochromatic objectives on an inverted LSM 5 microscope equipped with a laser scanning-disk confocal system (Zeiss). For life imaging of fungal hyphae, conidia were pre-grown on MM agar plate at 30 °C for 1 day. An agar piece containing mycelium was cut out and placed, upside down, onto an objective glass. To prevent drying-out of the agar/mycelium piece, 50 μl of MM was applied between the colony and the objective glass. After cells resumed growth (around one hour after the transfer) images were captured.

### Isolation and analysis of cell wall galactomannan and secreted galactomannoproteins

The isolation of cell wall (galacto)mannan was performed essentially as described by Bardalaye and Nordin [29] with minor modifications [30]. Monosaccharide analysis of the isolated (galacto)mannan fractions was performed by High-Performance Anion Exchange Chromatography (HPAEC) as described recently [30]. The Platelia assay (a quantitative assay for Gal*f* detection) was carried out as described [30]. A dot blot analysis using the L10 monoclonal anti-Gal*f*-antibody (1:10) [5] or ConA-labeled peroxidase (ConA-PO) (EY Laboratories, USA) were performed as described previously [30].

## Results

### Identification of two putative UDP-Gal*f* transporters in *A. niger* genome

In both *A. fumigatus* and *A. nidulans* UDP-Gal*f* transporter proteins have recently been identified [18, 19]. UDP-Gal*f* transporters belong to the family of nucleotide sugar transporters (NSTs) and the genomes of aspergilli contain at least 12 subfamilies of NSTs [15]. The indication for a possible role in UDP-Gal*f* biosynthesis of this class of NST was the chromosomal clustering with UgmA. UgmA (named GlfA in *A. fumigatus*) is a UDP-Gal*p* mutase which is required for the formation of UDP-Gal*f* [4, 31, 32]. The clustering of *ugmA* homologs with *ugtA* homologs is conserved among Pezizomycotina whose genome has been fully sequenced ([11], http://www.aspergillusgenome.org). Also in *A. niger*, UgmA (An02g08660) is clustered with a putative UDP-Gal*f* transporter (An02g08670) that we named UgtA. Interestingly, the genome of *A. niger* contains an additional close homolog of UgtA that we named UgtB (An06g00300). UgtB homologs were identified in genomes of nine other aspergilli (*Aspergillus acidus* CBS 106.47, *Aspergillus tubingensis* CBS 134.48, *Aspergillus kawachii*, *Aspergillus brasiliensis* CBS 101740, *Aspergillus carbonarius* ITEM 5010, *Aspergillus aculeatus* ATCC16872, *Aspergillus zonatus*, *Aspergillus wentii* DTO 134E9 and *Aspergillus glaucus* CBS 516.65) (http://www.aspergillusgenome.org/). The genome of *A. wentii* contains also a third homolog. As all these nine aspergilli also contain an UgtA homolog, (defined as such because homology and the genomic clustering with UgmA), it means that all these nine species have two Ugt paralogs (UgtA and UgtB). Phylogenetically, UgtA orthologs and UgtB orthologs cluster separately (Additional file 1: Figure S1). Note that *A. nidulans* and *A. fumigatus* only possess a single UDP-Gal*f* transporter, and deletion of this gene resulted in a Gal*f*-negative phenotype [18, 19].

*A. niger* UgtA and UgtB are proteins consisting of 399 and 339 amino acids, respectively, and share 67 % sequence identity between them. UgtA and UgtB are structurally conserved proteins belonging to the superfamily of nucleotide sugar transporters and predicted to contain 11 and 10 transmembrane domains, respectively (see Discussion).

### UgtA and UgtB have redundant function in biosynthesis of Gal*f*-glycoconjugates

**Fig. 1:**
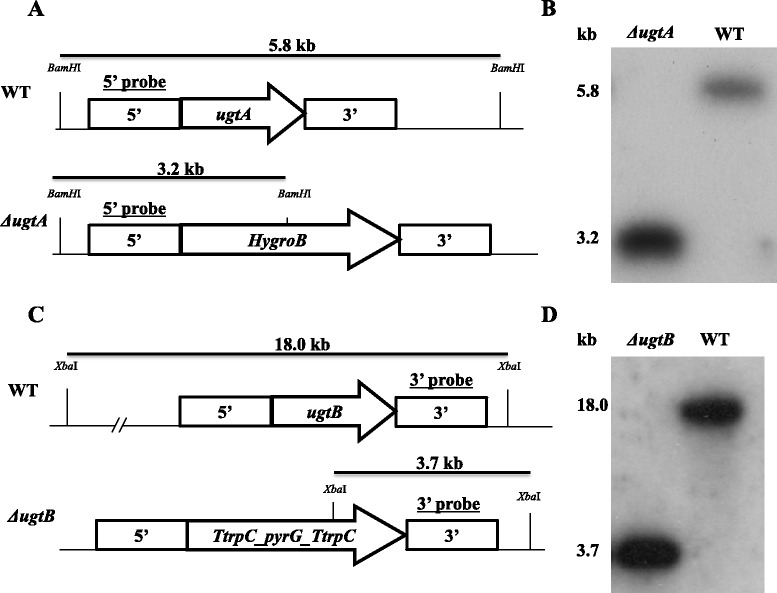
Gene deletion of *ugtA* and *ugtB* in *A. niger*. **a** and **c** Schematic representation of the strategy to disrupt *ugtA* (**a**) and *ugtB* (**c**) using the hygromycinB selection marker (HygroB) or the *pyrG* selection marker flanker by repeats of the trpC terminator regions (TtrpC). The 5’*ugtA* and the 3’*ugtB* probes used for hybridisation are indicated. Genomic DNA was digested with *BamH*I (UgtA blot) or *Xba*I (UgtB blot) and the length of the expected fragments is indicated. **b** and **d** Southern blots of genomic DNA after digestion and hybridisation with ^32^P-labelled probes. Approximate sizes of the bands based on DNA ladder (not shown) are indicated

To determine the function of UgtA and UgtB, deletion cassettes were constructed to delete the *ugtA* and *ugtB* genes individually, and to create the *ΔugtAΔugtB* double mutants described in Methods. Putative deletion mutants were subjected to Southern blot analysis and mutants with the proper deletion of the corresponding gene(s) were selected for further analysis (Fig. 1).

**Fig. 2:**
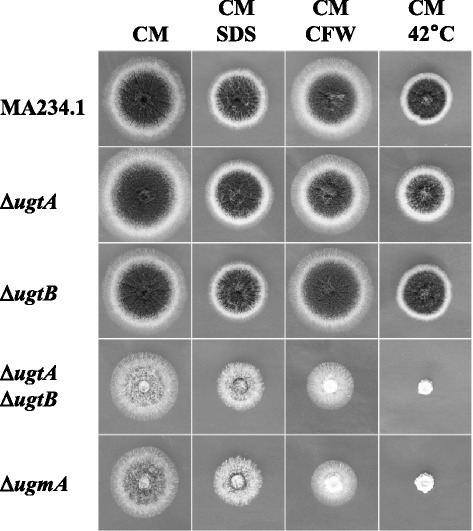
Phenotypic analysis of *ugt* mutants. Ten thousand spores of the indicated strains were spotted in the centre of a 9 cm Petri dish containing complete medium-agar supplemented with 0.0025 % SDS, or supplemented with 100 μg/ml CFW and incubated for three days at 30 °C. The mutant strains were also grown on complete medium-agar at 42 °C for 3 days

The *ΔugtA* and *ΔugtB* strains displayed no obvious phenotype when grown on plate, whereas the *ΔugtAΔugtB* double mutant showed a reduced growth and reduced sporulation phenotype (Fig. 2). Quantification of the numbers of spores indicated a ten-times reduction for both the *ΔugmA* and the *ΔugtAΔugtB* mutant (data not shown). The growth phenotype of the *ΔugtAΔugtB* double mutant is similar to that of the *A. niger ΔugmA* mutant (Fig. 2). Like those of the *ΔugmA* mutant, the hyphae of the *ΔugtAΔugtB* mutant are irregular in shape, and hyphal compartments are reduced in length (data not shown).

Deletion of *ugmA* results in an increased sensitivity towards Calcofluor White (CFW) and SDS. Both compounds are indicative for a compromised integrity of the cell wall [4, 33]. Similar to the *ΔugmA* mutant, the *ΔugtAΔugtB* mutant displays an increased sensitivity towards CFW and SDS (Fig. 2). Like the *ΔugmA* mutant, the *ΔugtAΔugtB* mutant also displays a strong growth defect at 42 °C (Fig. 2).

**Fig. 3:**
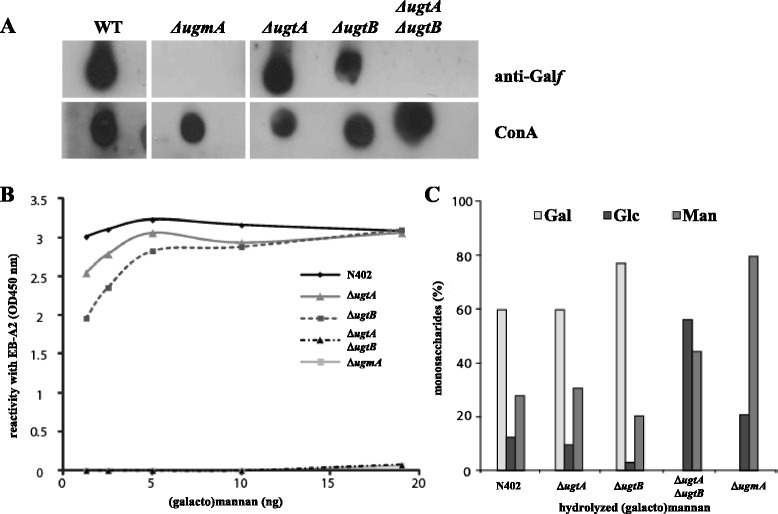
Analysis of Gal*f*-containing glycoconjugates in wildtype and Gal*f* mutants. **a** Dot blot assay to detect the presence of Gal*f* residues on secreted glycoconjugates from *A. niger* mutants. *A. niger* wild-type strain and Gal*f* mutants were grown to early stationary phase and cell-free medium was spotted on nitrocellulose filter paper. The blots were incubated with the anti-Gal*f* antibody (L10) to detect the presence of Gal*f* or incubated with ConA-PO to detect mannoproteins. **b** Platelia assay with anti-Gal*f* antibody EB-A2 was performed on indicated amounts of purified (galacto)mannan from indicated strains. **c** The percentage of the monosaccharides (Gal = galactose, Glc = glucose, Man = mannose) detected on HPAEC after hydrolysis of (galacto)mannan from indicated strains is shown. Figures shown are representative for at least two independent experiments

To assess Gal*f* biosynthesis in the *ΔugtAΔugtB* double mutant, medium samples of the wild-type strain N402 and the *ΔugmA*, *ΔugtA*, *ΔugtB* and *ΔugtAΔugtB* mutants were analysed for the presence of Gal*f* by a dot blot analysis using anti-Gal*f* antibody L10 [30]. As shown in Fig. 3a, no reactivity of L10 was detected towards the medium samples of the *ΔugtAΔugtB* double mutant, similar to that of the *ΔugmA* mutant. In contrast, Gal*f* was clearly present in the medium samples of both *ΔugtA* and *ΔugtB* strains. To determine the Gal*f* content quantitatively, galactomannan of the various strains was isolated [30] and their Gal*f* content established by detection with the anti-Gal*f* antibody EB-A2 in the Platelia assay (Fig. 3b). The data indicated that the galactomannan of both single Ugt mutants have a slightly lower Gal*f*-content compared to the wild-type strain, whereas the *ΔugtAΔugtB* mutant has no detectable Gal*f*, similar as the *ΔugmA* mutant. The isolated (galacto)mannan fractions were also subjected to hydrolysis and their monosaccharide content was subsequently determined by HPAEC (Fig. 3c), which confirmed the absence of Gal*f* in the polysaccharide fraction of the *ΔugtAΔugtB* and the *ΔugmA* mutants. These phenotypic analyses indicate that the transport activity of either UgtA or UgtB is sufficient to produce a galactomannan with wild-type properties, but that the lack of both transporters will prevent galactofuranosylation of the mannan backbone.

### Cellular localization of UgtA and UgtB

To localize UgtA and UgtB in *A. niger*, both proteins were C-terminally tagged with CFP or YFP using a GATEWAY based strategy. Both fusion genes were expressed from and integrated at their endogenous locus. The *A. oryzae pyrG* selection marker was flanked by *A. nidulans trpC* repeats inserted downstream of the *ugtA* or *ugtB* coding region. This strategy allowed integration of the fusion gene at the endogenous locus via homologous recombination and also allowed reuse of the *pyrG* marker because the marker can be efficiently removed by 5’FOA counter selection [24].

The constructs containing the CFP-tagged Gal*f* transporters (UgtA::CFP and UgtB::CFP) were transformed to *A. niger ku70* strain MA169.4 and uridine prototrophic transformants were purified. Proper recombination at either the *ugtA* or *ugtB* locus of the respective cassette was confirmed by Southern blot analysis (data not shown). To prove functionality of the fusion proteins, the UgtA-CFP and UgtB-CFP fusion constructs were also transformed to the *ΔugtB* and the *ΔugtA* strains, respectively. Again, proper integration of the tagged transporters was confirmed by Southern blot analysis (data not shown). Analysis of these transformants in which only one of the Gal*f*-transporters is present in a fluorescently-labelled form showed that the fusion proteins are fully functional as their growth was identical to the growth of the control strains (data not shown).

**Fig. 4:**
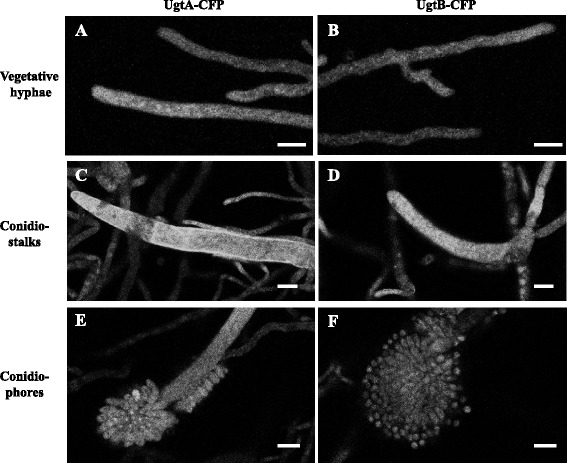
Subcellular localization of UgtA and UgtB in *A. niger*. Strain MH1.1 (UgtA::CFP) and MH2.1 (UgtB::CFP) were grown on MM-agar medium and analysed by fluorescence microscopy during vegetative growth (**a, b**) early conidiostalk formation (**c** and **d**) and during conidiospore formation (**e, f**). Bars represent 10 μm

Microscopic analysis of growing hyphal cells using confocal fluorescent microscopy confirmed the expected Golgi localization of both proteins. In both cases, a punctuated localization in the hyphal cells was observed, which is indicative for localization in Golgi equivalents in *A. niger* and other fungi [13, 14, 34]. Comparison of the fluorescence patterns of UgtA and UgtB to marker strains in which ER [12], secretory vesicles [35] or vacuoles (Ram, unpublished) were labelled clearly showed a different pattern. Comparison of the intensity signals of both UgtA-CFP and UgtB-CFP suggested higher amounts of UgtB-CFP in the Golgi membranes compared to UgtA. In general, the signals from strains expressing UgtA-CFP were weaker and more diffuse than these from strains expressing UgtB-CFP. To allow visualization of UgtA-CFP, the fluorescence picture shown in Fig. 4a was enhanced.

The expression and localization of UgtA-CFP and UgtB-CFP was also examined during asexual development. As shown in Fig. 4c to f, UgtA-CFP and UgtB-CFP are present in Golgi-like structure in conidiostalks and in young conidiospores. Enhancement of UgtA-CFP fluorescence was not necessary during conidiophores formation, indicating that the expression of UgtA is relative higher during conidiophore formation.

**Fig. 5:**
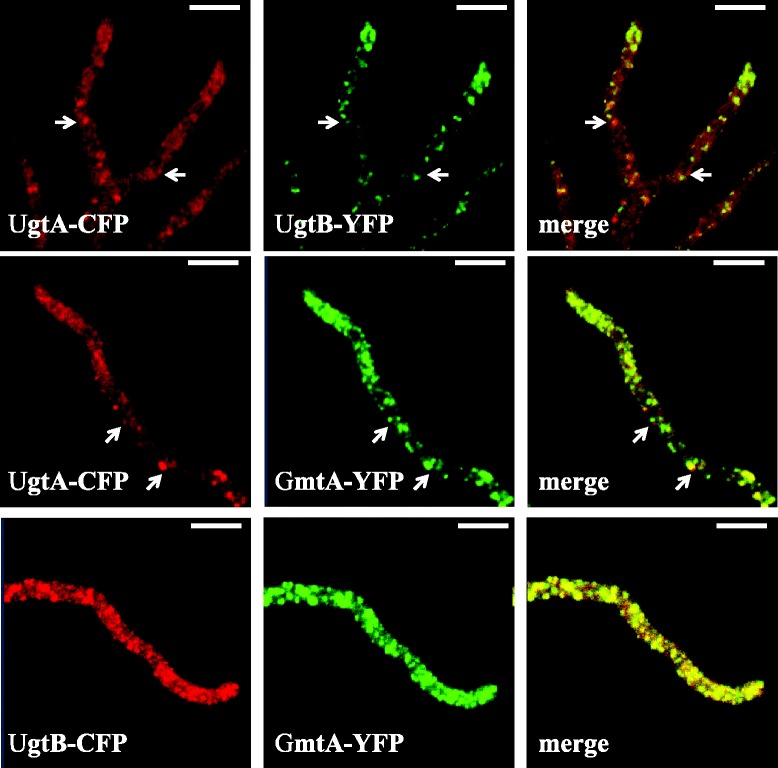
Co-localization studies of strain expressing differentially labeled nucleotide sugar transporters. Strains expressing UgtA::CFP and UgtB::YFP (JH24.3), UgtA::CFP and GmtA::YFP (JH22.3) and UgtB::CFP and GmtA::YFP (JH23.3) were grown and MM-agar plugs and imaged by confocal microscopy. Arrows point at UgtA spots. Images were false colored to red (CFP signal) and green (YFP signal) to improve contrast. Bars represent 10 μm

To analyse the possible co-localization of UgtA and UgtB and to look at co-localization of Gal*f* transporters (UgtA or UgtB) with a Golgi-localized GDP-mannose transporter (GmtA), several strains were constructed in which two nucleotide sugar transporters UgtA-CFP/UgtB-YFP; UgtA-CFP/GmtA-YFP; UgtB-CFP/GmtA-YFP were labelled with two different fluorescent proteins for analysis by fluorescence microscopy. Previously, we have shown the functionality of the YFP-tagged GDP-mannose transporter GmtA in *A. niger* [12]. It should be noted that the UgtA-CFP/GmtA-YFP and the UgtB-CFP/GmtA-YFP also contain the endogenous *ugtB* and *ugtA* genes, respectively, as well as the *gmtA* gene. In the UgtA-CFP/UgtB-YFP transformant, the signal of UgtB-YFP was stronger and quenched less quickly compared to the UgtA-CFP signal. The difference in intensity and quenching made it difficult to draw strong conclusions about the co-localization of both proteins. In general the UgtB-YFP and UgtA-CFP signals fluorescent images overlap, indicating co-localization of UgtA and UgtB in the Golgi. However, the presence of some clear UgtA spots (see arrows in Fig. 5) indicating that some Golgi equivalent seems to exist with a differential spatial distribution of UgtA and UgtB.

## Discussion

Over the past few years, genes and proteins involved in the biosynthesis of galactofuranose (Gal*f*)-containing glycoconjugates such as galactomannan, glycoproteins and glycolipids have been identified in fungal species. At least four essential enzymatic or transport steps are required; two enzymes for the synthesis of the sugar donor (UDP-Gal*f*), one transporter step for the translocation of the sugar donor from the cytosol, and finally the transfer of Golgi-localized UDP-Gal*f* to glycoconjugates via a glycosyltransferase [10]. The first step is the conversion of UDP-glucose to UDP-Gal which requires the presence of a UDP-glucose epimerase (referred to as Uge-activity). The second step involves the conversion of UDP-Gal into UDP-Gal*f* and is carried out by the enzyme named UDP-Gal*f* mutase (referred to as Ugm-activity). Transport of the UDP-Gal*f* from the cytosol to the Golgi requires specific UDP-Gal*f* transporters (referred to as Ugt-activity). The transferase activity is mediated by Golgi-localized UDP-Gal*f*transferases (referred to as Gfs-activity). The genes encoding the different enzymes or transporters have been identified in three Aspergillus species (*A. fumigatus, A. nidulans* and *A. niger*), based on reverse genetic approaches [10, 20, 36], genomics- and BLAST-based searches [10, 18, 19, 37, 38] or mutant screens [4, 29]. Studies in these *Aspergillus* species have shown that Gal*f* biosynthesis is important for maintaining cell wall integrity and virulence (in the case of *A. fumigatus*) [4, 6, 30].

Previous research in our group related to the identification of genes involved in Gal*f* biosynthesis was based on a screen for cell wall mutants with an induced expression of alpha-glucan synthase A (AgsA) [4]. AgsA is specifically induced in response to cell wall stress conditions and both the *ugeA* and the *ugmA* genes encoding the UDP-glucose-4- epimerase and the UDP-Gal*f* mutase, respectively, were identified via this screen [4, 29]. The screen for cell wall mutants with increased expression of *agsA* yielded 240 mutants which were all tested in detail for defects in Gal*f* biosynthesis as it was expected that also other genes involved in Gal*f* biosynthesis would lead to *agsA* induction. However, no other mutants besides the *ugeA* and *ugmA* mutants were identified. This study together with some unpublished results (see below) explain why additional mutants (e.g. in the UDP-Gal*f* transporter or UDP-Gal*f* transferase mutants) were not identified in the cell wall mutant screen. The present study clearly shows that *A. niger* contains two UDP-Gal*f* transporter genes with redundant functions. Inactivation by targeted deletion of either one of them did not result in reduced Gal*f* levels and consequently did not result in *agsA* induction. Genetic redundancy is also the reason why the gene encoding the UDP-Gal*f* transferase was not identified. The genome of *A. niger* contains three Gfs homologs and deletion of either *gfsA*, *gfsB* or *gfsC* did not result in *agsA* induction (Arentshorst and Ram, unpublished results). It should be noticed however that deletion of *gfsA* resulted in reduced levels of Gal*f* in a dot blot analysis (Arentshorst, Lagendijk and Ram, unpublished results), but this reduction was not sufficient to induce *agsA* expression.

The redundancy of Gal*f* transporters was noticed in nine Aspergillus species in addition to *A. niger* that are present in the AspGD database (http://www.aspergillusgenome.org/). Six of these species belong to the Aspergillus section nigri (*A. acidus* CBS 106.47, *A. tubingensis* CBS 134.48, *A. kawachii*, *A. brasiliensis* CBS 101740, *A. carbonarius* ITEM 5010, *A. aculeatus* ATCC16872), but *A. zonatus*, *A. wentii* DTO 134E9 and *A. glaucus* CBS 516.65 are more distantly related and belong to other phylogenetic groups [30]. The presence of these homologs in distantly related groups that also show high level of synteny, suggests that the duplication has been a rather early event in evolution and that the second gene has been lost in many species also in the nigri section. E.g. closely related species like *A. terreus* have only one copy. It is also interesting to note that the presumed loss of one of two genes is not random. The UgtA homologs (defined by the genomic clustering of this transporter with the *ugmA* gene ([18]) are always present while the *ugmB* gene can be absent from the genome. The reason for this preferential presence of UgtA is not clear as we could not detect an effect on growth of the deletion of *ugtA* in *A. niger*.

The UgtA and UgtB proteins are predicted to be 399 and 339 amino acid residues long, respectively. We slightly modified the gene model which is present in AspGD for the *ugtB* gene. The first predicted intron is likely to be 18 nucleotides shorter thereby including six amino acids that are also predicted to be present in all UgtB homologs. In addition, the addition of these six amino acids also improved alignment with UgtA homologs. Both UgtA and UgtB are predicted to be nucleotide sugar transporters based on the presence of pfam motif03151 (triose phosphate transporters). Alignment of the protein sequences of UgtA and UgtB revealed that the difference in length between UgtA and UgtB is mainly caused by a shorter C-terminal region of the UgtB protein (Additional file 2: Figure S2). The membrane topology of nucleotide sugar transporters (NST) has been predicted to comprise between 6, 8 or 10 transmembrane domains, linked by hydrophilic loops at both sites of the membrane. To data, all NST are predicted to have an even number of transmembrane domains in which the N and C-termini of the NST are located in the cytosol. The only exception is the *A. fumigatus* UgtA/GlfB protein. Alignment of *A. niger* UgtA and UgtB proteins to the *A. fumigatus* UgtA/GlfB protein suggested that the *A. niger* UgtA protein also comprises 11 transmembrane domains. In comparison with other, more distantly related NSTs, UgtA seems to possess an additional TM domain in the C-terminal part of the protein (amino acid residues 358–378 (see Engel [18]). Alignment of the *A. niger* UgtB protein with *A. niger* UgtA and *A. fumigatus* UgtA/GlfB suggests that UgtB contains ten TM, and lacks the most C-terminal TM domain present in UgtA (Additional file 2: Figure S2). Although not experimentally validated, we propose that UgtB has ten TM domains and in comparison to UgtA proteins lacks the most C-terminal TM domain.

The localization studies of the two Gal*f* transporters and the GDP-mannose transporter suggest that the proteins co-localize in Golgi equivalents. Previous data in *A. nidulans* have indicated that the GDP-mannose transporter in *A. nidulans* does not co-localize with CopA [11]. CopA is a conserved component of the coat-protein complex I (COPI) coatomer complex required for retrograde transport between Golgi compartments and between Golgi and ER [40] and considered as an early Golgi marker. Several additional markers for early Golgi compartments (SedV, RerA and RabO) or late-Golgi compartments (mRFP-PH^OSBP^, and TlgB) have recently been identified in *A. nidulans* [34, 41]. Yet another aspect that requires further attention is the mechanism by which these Gal*f* transporters are retained in Golgi cisternae. Lysine motifs at the C-terminus of the GDP-mannose transporter have been implicated in their Golgi-retrieval [16], but such a motif was not detected in UgtA or UgtB. A role for the C-terminal part of UgtA or UgtB in a general mechanism for retrieval seems unlikely as the C-terminal parts of both proteins are very different. The GFP-tagged versions of UgtA and UgtB created in this study, provide starting tools for future studies on mutants or mutations in UgtA or UgtB that possibly affect their localization in the Golgi.

## Conclusions

*A. niger* possesses not a single, but two genes encoding UDP-Gal*f* transporters. Both proteins are localized in the Golgi and contribute to the transport of UDP-Gal*f* over the Golgi membrane. Deletion of only one of the two transporter genes, did not result in obvious growth defects or reduced levels of Gal*f* in the cell wall or on glycoproteins indicating that the two proteins have redundant functions. The overlapping function of the two proteins was further shown by simultaneous deletion of the two transporter genes which resulted is the absence of Gal*f*-containing glycoconjugates.

## Additional files

Additional file 1: Figure S1. Homology tree of UgtA and UgtB homologs in Aspergilli. Protein sequence homologous to *A. niger* UgtA or *A. niger* UgtB were extracted from the AspGD database and aligned using DNAman. % of amino acid identity is given. (PPTX 303 kb) Additional file 2: Figure S2. Amino acid alignment of *A. niger* UgtA and UgtB proteins with UgtA/GlfB of *A. fumigatus*. Transmembrane domains predicted in UgtA/GlfB and homologous region in the *A. niger* proteins are highlighted. (DOCX 16 kb)

## Abbreviations

Gal*p*: Galactopyranose
Gal*f*: Galactofuranose
UDP: Uridine diphosphate
GDP: Guanine diphosphate
Ugt: UDP-Gal*f* transporter
GFP: Green fluorescent protein
CFP: Cyan fluorescent protein
YFP: Yellow fluorescent protein
ER: Endoplasmic reticulum
HPAEC: High-performance anion-exchange chromatography
5’FOA: 5’fluoroorotic acid
