# Supplementary figures and images for: Identification and functional analysis of two Golgi-localized UDP-galactofuranose transporters with overlapping functions in Aspergillus niger
Source: BMC Microbiol. 2015 Nov 2;15:253. doi: 10.1186/s12866-015-0541-2 (PMC4630932; doi:10.1186/s12866-015-0541-2)

## Slide 1
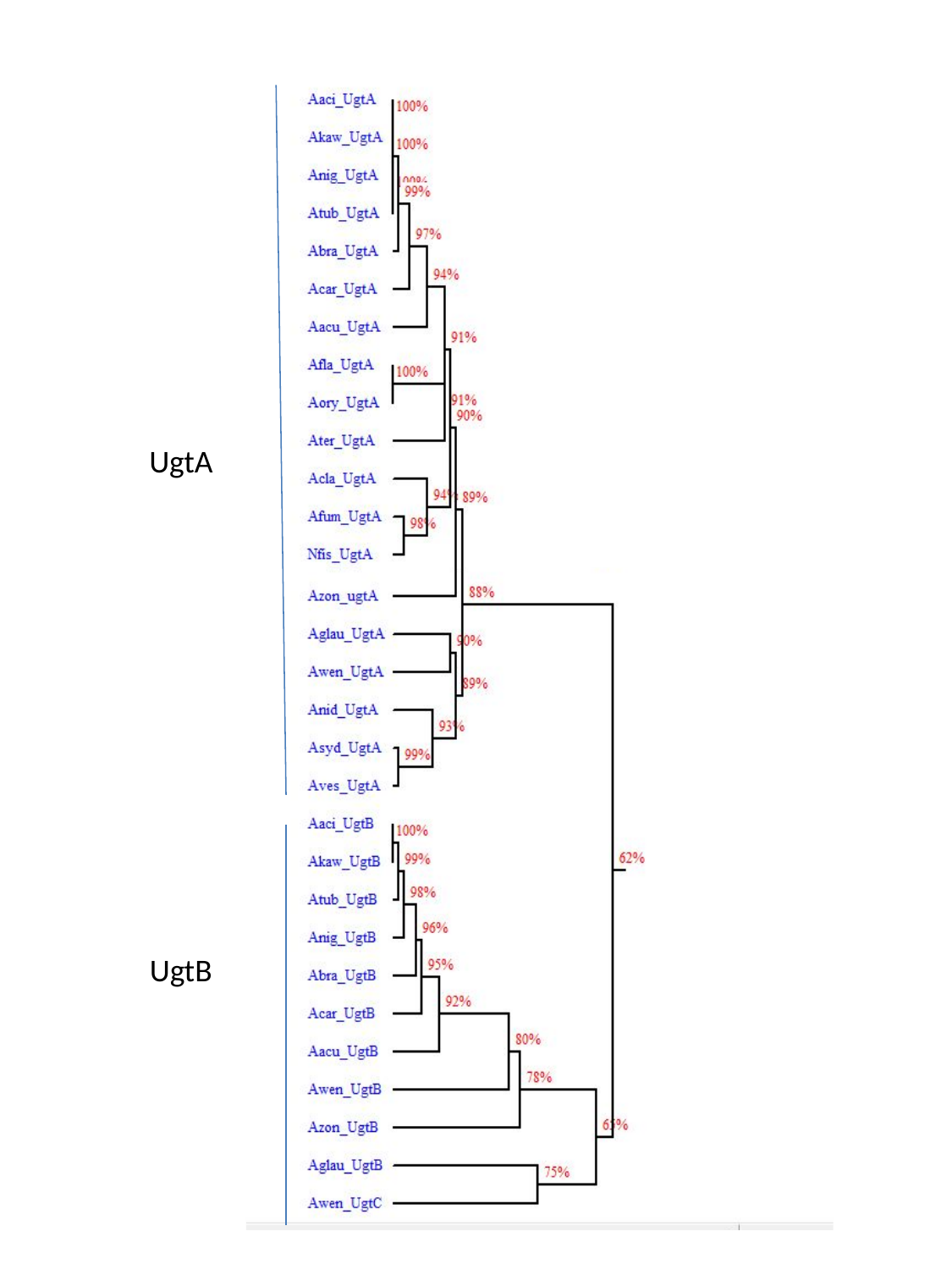

UgtA
UgtB

Supplement: Additional file 1: Figure S1. — Homology tree of UgtA and UgtB homologs in Aspergilli. Protein sequence homologous to A. niger UgtA or A. niger UgtB were extracted from the AspGD database and aligned using DNAman. % of amino acid identity is given. (PPTX 303 kb) [file 12866_2015_541_MOESM1_ESM.pptx]
