# Supplementary material for: Identification and functional analysis of two Golgi-localized UDP-galactofuranose transporters with overlapping functions in Aspergillus niger
Source: BMC Microbiol. 2015 Nov 2;15:253. doi: 10.1186/s12866-015-0541-2 (PMC4630932; doi:10.1186/s12866-015-0541-2)
Supplement: Additional file 2: Figure S2. — Amino acid alignment of A. niger UgtA and UgtB proteins with UgtA/GlfB of A. fumigatus. Transmembrane domains predicted in UgtA/GlfB and homologous region in the A. niger proteins are highlighted. (DOCX 16 kb) [file 12866_2015_541_MOESM2_ESM.docx]

Anig_UgtA -MSEGEKARTSGEVSRPEPTLPTVNPAVDKAEPPKPAFHPAVYVGVWITLSSSVILFNKH 59

Anig_UgtB --------------MAAKSQLPTVNTPVSA-EPSKAAMHPAFYIATWIALSSGVIIFNKW 46

Afum_UgtA MSNEGEKARVSGEVSRPEPTLPTVNPAVEKSEPSKPTFHPAVYVSLWIALSSSVILFNKH 60

***** * ** * . *** *. **.*** **.***

TMD I

Anig_UgtA ILDYAQFRFPIILTTWHLAFATFMTQLLARTTTLLDGRKTVKMTGRVYLRAIVPIGLFFS 119

Anig_UgtB ILHTAGFSFPLFLTTWHLVFATIMTRLMARFTTLLDSRHQVPMTSRVYMRAIVPIGAFFS 106

Afum_UgtA ILDYAQFRFPIILTTWHLAFATFMTQVLARTTTLLDGRKTVKMTGRVYLRAIVPIGLFFS 120

** * * **. ****** *** **...** ***** *. * ** ***.******* ***

TMD II TMD III

Anig_UgtA LSLICGNVTYLYLSVAFIQMLKATTPVAVLFATWGMGMAPVNLKVLMNVSIIVLGVIIAS 179

Anig_UgtB LSLICGNLAYLYLSVSFIQMLKATNSVATLLATWAMGIAPVKLSLLGNISFIVLGVIIAS 166

Afum_UgtA LSLICGNVTYLYLSVAFIQMLKATTPVAVLLATWAMGMAPVNLKVLFNVAVIVIGVVIAS 180

*******..******.********. ** * *** **.*** * .* *.. **.**.***

TMD IV TMD V

Anig_UgtA FGEIRFVFIGFLFQLGGIVFEATRLVMVQRLLSSAEYKMDPLVSLYYFAPVCAVMNGVTA 239

Anig_UgtB IGEIKFTMIGFICQFFATIFESVRLVMVQRLLSSAEFKMDPLVSLYYFAPACAVMNAVVT 226

Afum_UgtA FGEIKFVFIGFLFQIGGIVFEATRLVMVQRLLSSAEFKMDPLVSLYYFAPVCAVMNGVTA 240

***.* ***. * .**. *************.************* ***** * .

TMD VI TMD VII

Anig_UgtA LFLEVPNLTMGHIYNVGVWTLLANAVVAFLLNVSVVFLIGKTSSLVMTLCGVLKDILLVA 299

Anig_UgtB AVVELPTLHMSDIYQLGMGTLFLNAAVAFGLNVAVVFLIGKTSALVLTLSGVLKDILLVV 286

Afum_UgtA LFVEVPNLTMGHIYNVGIWTLLANAVVAFLLNVSVVFLIGKTSSLVMTLCGVLKDILLVA 300

.*.*.* * **..*. ** ** *** ***.*********.**.**.*********

TMD VIII TMD XI

Anig_UgtA ASMMIWQTPVTLTQFFGYSIALVGLVYYKLGGDKIKEYTGQANRAWAEYGVNHPAQRKFI 359

Anig_UgtB ASMVIFRDPVTPLQAFGYAIALGGLVYYKLGRDGVNNLLAQVRYQVLGGPRPEN------ 339

Afum_UgtA ASMMIWQTPVTPLQFFGYSIALIGLVYYKLGGDKIREYAGQANRSWAEYGANHPAQRKSI 360

***.* . *** * ***.*** ******** * . *

TMD X

Anig_UgtA VFGAILLIFFLLMGSMAPSYAPEQVASVKGMLGGATAGNA 399

Anig_UgtB ---------------------------------------- 339

Afum_UgtA IIGAVVLIFFLLIGSMAPSYAPESVDKVKGMLGGATAGNA 400

TMD XI
